# Supplementary material for: Could an event of extreme drought (2019-2020) affect the feeding ecology of Bubo magellanicus (Gmelin 1788) (Strigiformes: Strigidae) in a Mediterranean region of Chile?
Source: PeerJ. 2023 May 1;11:e15020. doi: 10.7717/peerj.15020 (PMC10158770; doi:10.7717/peerj.15020)
Supplement: Supplemental Information 1 [file peerj-11-15020-s001.docx]

Supplementary materials

1.- Frequency of occurrence (%F)

$$F=\frac{ni}{N}*100$$

Where $ni$= number of pellets of the species$i$, $pi=$ mean mass of the species $i$, and $N$ = the total of pellets analysed.

2.- Percentage of biomass (%B)

$$B=\frac{pi*ni}{Mass total}*100$$

Where $ni$= number of pellets of the species$i$, $pi=$ mean mass of the species $i$, and $N$ = the total of pellets analysed.

3.- Shannon-Wiener index (H’)

$$H'= -\sum_{i=1}^{S} pi*pi$$

where $pi=$ the proportion of prey units of the species $i$ in relation to the total number of prey units ($\frac{ni}{N})$

4.- Pielou index (J´)

$$J'=\frac{H'}{H' max}$$

Where $H'max= ln(s)$ and $H'=$ Shannon-Wiener index.

5.- Levins index (B)

$$B= \frac{1}{\sum_{i=1}^{n} {pi}^{2}}$$

Where $pi=$ is the relative occurrence of a prey unit in relation to the total of prey units.

6.- Levins standardised index (Bsta)

$$B_{STA}=\frac{(Bobs \pm B min)}{(Bmax \pm Bmin)}$$

Where $B$obs is the trophic niche breadth observed, $B$min is equal to 1, and $B$max is the breadth of the maximum possible trophic niche.
